# Autoimmune Epilepsy: Some Epilepsy Patients Harbor Autoantibodies to Glutamate Receptors and dsDNA on both Sides of the Blood-brain Barrier, which may Kill Neurons and Decrease in Brain Fluids after Hemispherotomy

**DOI:** 10.1080/17402520400001736

**Published:** 2004

**Authors:** Yonatan Ganor, Hadassa Goldberg-Stern, Dina Amrom, Tally Lerman-Sagie, Vivian I. Teichberg, Dori Pelled, Anthony H. Futerman, Bruria Ben Zeev, Michael Freilinger, Denis Verheulpen, Patrick Van Bogaert, Mia Levite

**Affiliations:** 1 Department of Neurobiology The Weizmann Institute of Science Rehovot Israel; 2 Epilepsy Center Schneider Children's Medical Center of Israel Petah Tiqwa Israel; 3 Sackler Faculty of Medicine Tel Aviv University aviv Israel; 4 Department of Child Neurology Hôpital Français-ULB Brussels Belgium; 5 Pediatric Neurology Unit Wolfson Medical Center Holon Israel; 6 Department of Biological Chemistry The Weizmann Institute of Science Rehovot Israel; 7 Department of Child Neurology and Epilepsy Sheba Medical Center hashomer Israel; 8 Department of Pediatrics University Hospital of Vienna Austria; 9 Department of Pediatric Neurology ULB-Hopital Erasme Brussels Belgium

## Abstract

*Purpose*: Elucidating the potential contribution of specific autoantibodies (Ab's) to the etiology and/or pathology of some human epilepsies. *Methods*: Six epilepsy patients with Rasmussen's encephalitis (RE) and 71 patients with other epilepsies were tested for Ab's to the –B— peptide (amino acids 372-395) of the glutamate/AMPA subtype 3 receptor (GluR3B peptide), double-stranded DNA (dsDNA), and additional autoimmune disease-associated autoantigens, and for the ability of their serum and cerebrospinal-fluid (CSF) to kill neurons. *Results*: Elevated anti-GluR3B Ab's were found in serum and CSF of most RE patients, and in serum of 17/71 (24%) patients with other epilepsies. In two RE patients, anti-GluR3B Ab's decreased drastically in CSF following functional-hemispherotomy, in association with seizure cessation and neurological improvement. Serum and CSF of two RE patients, and serum of 12/71 (17%) patients with other epilepsies, contained elevated anti-dsDNA Ab's, the hallmark of systemic-lupus-erythematosus. The sera (but not the CSF) of some RE patients contained also clinically elevated levels of –classical— autoimmune Ab's to glutamic-acid-decarboxylase, cardiolipin, β2-glycoprotein-I and nuclear-antigens SS-A and RNP-70. Sera and CSF of some RE patients caused substantial death of hippocampal neurons. *Conclusions*: Some epilepsy patients harbor Ab's to GluR3 and dsDNA on both sides of the blood-brain barrier, and additional autoimmune Ab's only in serum. Since all these Ab's may be detrimental to the nervous system and/or peripheral organs, we recommend testing for their presence in epilepsy, and silencing their activity in Ab-positive patients.


*Clinical & Developmental Immunology*, September/December 2004, Vol. 11 (3/4), pp. 241–252

Taylor & Francis healthsciences

### Autoimmune Epilepsy: Some Epilepsy Patients Harbor Autoantibodies to Glutamate Receptors and dsDNA on both Sides of the Blood–brain Barrier, which may Kill Neurons and Decrease in Brain Fluids after Hemispherotomy

YONATAN GANOR[a], HADASSA GOLDBERG-STERN[b,c], DINA AMROM[d], TALLY LERMAN-SAGIE[e,c], VIVIAN I. TEICHBERG[a], DORI PELLED[f], ANTHONY H. FUTERMAN[f], BRURIA BEN ZEEV[g,c], MICHAEL FREILINGER[h], DENIS VERHEULPEN[i], PATRICK VAN BOGAERT[i] and MIA LEVITE[a,c,*]

[a]*Department of Neurobiology, The Weizmann Institute of Science, Rehovot, Israel;* [b]*Epilepsy Center, Schneider Children's Medical Center of Israel, Petah Tiqwa, Israel;* [c]*Sackler Faculty of Medicine, Tel Aviv University, Tel Aviv, Israel;* [d]*Department of Child Neurology, Hôpital Français-ULB, Brussels, Belgium;* [e]*Pediatric Neurology Unit, Wolfson Medical Center, Holon, Israel;* [f]*Department of Biological Chemistry, The Weizmann Institute of Science, Rehovot, Israel;* [g]*Department of Child Neurology and Epilepsy, Sheba Medical Center, Tel Hashomer, Israel;* [h]*Department of Pediatrics, University Hospital of Vienna, Austria;* [i]*Department of Pediatric Neurology, ULB-Hopital Erasme, Brussels, Belgium*

*Purpose*: Elucidating the potential contribution of specific autoantibodies (Ab's) to the etiology and/or pathology of some human epilepsies.
*Methods*: Six epilepsy patients with Rasmussen's encephalitis (RE) and 71 patients with other epilepsies were tested for Ab's to the "B" peptide (amino acids 372–395) of the glutamate/AMPA subtype 3 receptor (GluR3B peptide), double-stranded DNA (dsDNA), and additional autoimmune disease-associated autoantigens, and for the ability of their serum and cerebrospinal-fluid (CSF) to kill neurons.
*Results*: Elevated anti-GluR3B Ab's were found in serum and CSF of most RE patients, and in serum of 17/71 (24%) patients with other epilepsies. In two RE patients, anti-GluR3B Ab's decreased drastically in CSF following functional-hemispherotomy, in association with seizure cessation and neurological improvement. Serum and CSF of two RE patients, and serum of 12/71 (17%) patients with other epilepsies, contained elevated anti-dsDNA Ab's, the hallmark of systemic-lupus-erythematosus. The sera (but not the CSF) of some RE patients contained also clinically elevated levels of "classical" autoimmune Ab's to glutamic-acid-decarboxylase, cardiolipin, β2-glycoprotein-I and nuclear-antigens SS-A and RNP-70. Sera and CSF of some RE patients caused substantial death of hippocampal neurons.
*Conclusions*: Some epilepsy patients harbor Ab's to GluR3 and dsDNA on both sides of the blood–brain barrier, and additional autoimmune Ab's only in serum. Since all these Ab's may be detrimental to the nervous system and/or peripheral organs, we recommend testing for their presence in epilepsy, and silencing their activity in Ab-positive patients.

*Keywords*: Antibodies; Autoimmunity; DNA; Epilepsy; GluR3; Rasmussen's encephalitis

### INTRODUCTION

Epilepsy is a major health problem, affecting ~1% of the world population. There are many different types of epilepsy, a substantial number with unknown etiology. Moreover, ~20–30% of all epilepsy patients are completely refractory to all anti-epileptic drugs (AEDs) and suffer from a low quality of life. Can autoimmunity contribute to the etiology and/or pathology of some epilepsies ? (Rogers *et al.*, 1994; Levite and Hermelin, 1999; Levite *et al.*, 1999; Wiendl *et al.*, 2001; Levite and Hart, 2002; Mantegazza *et al.*, 2002; Xiong *et al.*, 2003) and reviewed by Levite (2002).

Originally, an autoimmune process linked to specific autoantibodies (Ab's) to the glutamate/α-amino-3-hydroxy-5-methyl-4-isoxazolepropionic acid (AMPA) receptor subtype 3 (GluR3) was suggested to underlie only Rasmussen's encephalitis (RE), a progressive catastrophic epileptic encephalopathy of unknown etiology, causing damage primarily to one cerebral hemisphere, and characterized by intractable unilateral focal seizures, progressive neurological deficits, and variable intellectual impairment (Rasmussen *et al.*, 1958). Hemispheric atrophy, loss of cortical neurons, and chronic inflammatory changes are characteristic of RE histopathology.

*Corresponding author. Address: Department of Neurobiology, The Weizmann Institute of Science, Rehovot 76100, Israel. Tel.: +972-8-9342315; +972-54-4244814. Fax: +972-8-9344131. E-mail: mia.levite@weizmann.ac.il

ISSN 1740-2522 print/ISSN 1740-2530 online © 2004 Taylor & Francis Ltd
DOI: 10.1080/17402520400001736

Most patients with RE are unresponsive to conventional AEDs, and the only recommended therapeutic approach is surgical hemispherotomy, which either removes substantial parts of the affected hemisphere or disconnects the hemispheres while removing a minimal amount of cortical structures (functional hemispherotomy) (Villemure and Mascott, 1995).

The suggestion that the development of RE may result from the activity of deleterious autoimmune components in general, and specific anti-GluR3 Ab's in particular, was based primarily on the development of seizures and histopathologic features mimicking RE in two (out of five) rabbits immunized with a 212 amino-acid (aa) long extracellular domain of GluR3 (aa 245–457) (Rogers *et al.*, 1994), the finding of anti-GluR3 Ab's in some RE patients (Rogers *et al.*, 1994), and the beneficial effect of immunomodulatory treatment in a small number of RE patients by repeated plasma exchange (Rogers *et al.*, 1994; Andrews *et al.*, 1996) or immunoglobulin immunoabsorption (Antozzi *et al.*, 1998), causing a transient reduction in seizure frequency and improvement in neurological function, in correlation with reduced serum titers of anti-GluR3 Ab's.

In recent years, it has become clear that anti-GluR3 Ab's are present in some patients with other types of epilepsy, and thus that they are by no means specific or restricted to RE (Wiendl *et al.*, 2001; Mantegazza *et al.*, 2002). Specifically, anti-GluR3 Ab's were found in some patients with various types of epilepsy, suffering primarily from non-inflammatory focal epilepsy (Wiendl *et al.*, 2001), or from severe, early-onset and intractable seizures (Mantegazza *et al.*, 2002). Further studies and findings suggested that anti-GluR3 Ab's may indeed play a pathogenic role in epilepsy, since they can: (a) bind brain cells and structures (Levite *et al.*, 1999; Whitney and McNamara, 2000; Frassoni *et al.*, 2001; Bernasconi *et al.*, 2002), (b) activate ionotropic GluR's and evoke ion currents, acting like a "novel" glutamate agonist (Twyman *et al.*, 1995; Levite *et al.*, 1999; Koustova *et al.*, 2001), (c) kill neurons and glia cells via excitotoxicity—a fatal overactivation of the glutamate receptors (Levite *et al.*, 1999; Koustova *et al.*, 2001), alike that caused by excess glutamate in various pathological situations (Olney, 1990; Choi, 1992), and (d) activate the destructive complement system within the central nervous system (CNS), causing at first death of astrocytes, and then death of neurons (He *et al.*, 1998; Whitney and McNamara, 2000). In this context it is of interest to mention that the serial recruitment of all the complement factors in the CNS was recently shown to induce seizures (Xiong *et al.*, 2003).

In addition to all the above, mice immunized with the short GluR3-derived peptide: aa 372–395, termed GluR3B peptide, believed to be the major antigenic epitope of the excitotoxic anti-GluR3 Ab's (Rogers *et al.*, 1994; Twyman *et al.*, 1995), developed a brain pathology (Levite and Hermelin, 1999; Levite *et al.*, 1999), with features partially resembling those found in RE patients.

Taken together, these different types of observations suggest that anti-GluR3 Ab's have the ability to cause neuronal activation and death in the CNS, which could in principal lead to the outburst of epileptic seizures. Despite all that, the direct evidence that this occurs *in vivo* in epilepsy patients is still misssing and a great deal remains to be explored for reaching an understanding of the role of anti-GluR3 Ab's in epilepsy. Among the issues not studied thus far are whether the cerebrospinal fluid (CSF) levels of anti-GluR3B Ab's correlate with seizure frequency, whether human anti-GluR3B rich serum or CSF have the ability to kill neurons, and whether RE patients and potentially other epilepsy patients harbor in addition to the anti-GluR3 Ab's and other anti-glutamate receptor Ab's (Takahashi *et al.*, 2003), also elevated levels of other autoimmune Ab's of known pathogenic activity in the periphery and CNS. These latter Ab's could include: anti-double-stranded (ds) DNA Ab's, found primarily in systemic lupus erythematosus (SLE); anti-glutamic acid decarboxylase (GAD) Ab's, found primarily in insulin-dependent diabetes mellitus (IDDM) and in stiff person syndrome; anti-cardiolipin and anti-β2 glycoprotein I (β2GPI) Ab's, found primarily in anti-phospholipid syndrome (APS); and anti-nuclear Ab's found primarily in SLE and Sjögren's syndrome.

In the present study, we have investigated some of these still open issues.

### METHODS

### Patient Population

#### *Rasmussen's Encephalitis*

Serum was collected from six RE patients (two males): two from Sheba Medical Center, Israel; one from Wolfson Medical Center, Israel; one from Centre Hospitalier Francois Rabelais, Belgium; one from ULB-Hopital Erasme, Belgium; and one from University Hospital of Vienna, Austria. CSF was obtained from four of these RE patients. The mean age of the RE patients was 10.4 years (95% CI's 4.1–16.6 years). Diagnosis of RE was performed by the respective clinicians, and was based on the presence of several RE clinical characteristics, some of which detected by: computerized tomography (CT), magnetic resonance imaging (MRI), electroencephalography (EEG), single-photon emission scan tomography (SPECT), magnetic resonance spectroscopy (MRS) and [18F] fluorodeoxyglucose positron emission tomography (FDG-PET).

#### *Patients with other non-RE Types of Epilepsy*

Serum was obtained from 71 consecutive age-matched epilepsy patients during their routine visit at the Pediatric Epilepsy Center at Schneider Children's Medical Center of Israel. These patients were classified on the basis of the clinical and electrographic features of the International League Against Epilepsy classification (Proposal for revised classification of epilepsies and epileptic syndromes, 1989), into 51 with partial epilepsy (23 males, mean age 12.1 years; 95% CI's 10.3–13.9 years) and 20 with generalized epilepsy (12 males, mean age 10.4 years; 95% CI's 7.5–13.2 years).

Written informed consents for sampling blood and/or CSF were obtained from all patients or their parents/guardians, upon approval by the Helsinki Committee at their local institution.

### *Controls*

Serum (49 samples) and CSF (seven samples) of neurologically intact non-epileptic individuals were also studied (26 males, mean age 7.2 years; 95% CI's 5.5–8.9 years).

### Detection of Anti-GluR3B and Anti-dsDNA Ab's

The levels of anti-GluR3B Ab's in sera and CSF of epilepsy patients were tested by ELISA, as developed and described previously (Levite and Hermelin, 1999; Levite *et al.*, 1999), measuring in parallel the specific binding of each serum or CSF to the GluR3B peptide (NEYERFVPFSDQQISND-SSSSENR, synthesized at the Weizmann Institute of Science) and the non-specific binding to bovine serum albumin (BSA; Sigma). Specific anti-GluR3B Ab's in each individual was calculated as (specific binding to GluR3B—nonspecific binding to BSA).

The levels of anti-dsDNA Ab's were tested by ELISA, using plates coated with Lambda phage dsDNA (Worthington Biochemical Corporation, Lakewood, NJ, USA) as previously described (Eilat *et al.*, 2001).

### Detection of Ab's Directed against GAD, Cardiolipin, β2GPI, RNP-70 and SS-A

The Ab's against GAD, cardiolipin, β2GPI, RNP-70 and SS-A were measured by commercial ELISA diagnostic kits used routinely in hospitals worldwide. The clinical evaluation was performed as instructed by the respective manufacturer, which supplied the negative and positive controls for each Ab type, and the exact cutoff values above which a patient is considered positive. Specifically, the diagnostic kits and the clinical cutoffs were as follows: anti-GAD IgG diagnostic kit (Isletest-GAD; Biomerica, Newport Beach, CA, USA), anti-GAD Ab's level above 1.0 "GAD value" are considered clinically positive, and levels of 1.0–1.05 as borderline; anti-cardiolipin and anti-β2GPI diagnostic kits (ORG515 and ORG521; Orgentech Diagnostika GmbH, Mainz, Germany), anti-cardiolipin IgG levels above 10.0 U/ml and anti-β2GPI levels (both IgM and IgG) above 8.0 U/ml are considered clinically elevated, and levels of 5.0–8.0 U/ml are borderline; anti-RNP-70 and anti-SS-A IgG diagnostic kits (Medizym anti-RNP and Medizym anti-SS-A; Medipan Diagnostica, Selchow, Germany), anti-RNP Ab's level above 15.0 U/ml and anti-SS-A Ab's levels above 10.0 U/ml are considered clinically elevated.

### Immunostaining of Anti-nuclear Ab's (ANA)

ANA immunofluorescence staining, using commercial slides coated with fixed human epithelioma type 2 (HEp-2) cells (Immuno Concepts, USA), and fluorescein isothiocyanate (FITC)-conjugated anti-human IgG antibody (The Binding Site, UK) were performed according to the manufacturer's instructions.

### Evaluation of Intrathecal Ab Production

An IgG index was calculated (Link and Tibbling, 1977; Lefvert and Link, 1985) as follows: IgG index = (CSF IgG/serum IgG)/(CSF albumin/serum albumin). An IgG index $> 0.7$ is accepted as an indication for the intrathecal Ab's production (Link and Tibbling, 1977; Lefvert and Link, 1985).

### Measurements of Neuronal Death Caused by Serum or CSF of RE Patients

Cultures of rat hippocampal neurons (7–10 days old) were grown as described (Brann *et al.*, 2002). Serum or CSF (diluted 1:10) of RE patients and of age-matched neurologically healthy control individuals were first purged (by repetitive filtration through 30–50 kDa vivaspin filters, as described by Levite and Hermelin (1999), Levite *et al.* (1999)) of glutamate and of additional small molecules (to eliminate their potential contribution to neuronal death), and then added to the neuronal cultures. After 18 h (37°C, humidified incubator), live and dead neurons were distinguished using a Live/Dead® viability/cytotoxicity kit (Molecular Probes, USA) as described (Brann *et al.*, 2002).

### Functional Hemispherotomy

A functional peri-insular hemispherotomy was performed according to Villemure's technique (Villemure and Mascott, 1995). This operation, a variant of functional hemispherotomy for epilepsy, allows complete disconnection of the diseased hemisphere, while removing certain brain regions, including the temporal opercula and white matter, cortex, temporal gyrus, amygdala, 5–10 mm insular cortex, and frontobasal cortex and white matter (Villemure and Mascott, 1995). Thus, an extensive disconnection of the epileptic area was achieved, but most of the inflammatory parenchyma was left in the cranium with functional vascularisation.

### The Neurological Condition of RE Patients No. 1 and 6, Before and After Functional Hemispherotomy

RE patient no. 1 (female, age of nine years) was referred for surgery following 20 months of disease progression, after reaching a stage of intractable epilepsy with loss of the right hand function. She underwent functional peri-insular hemispherotomy according to the Villemure's technique (Villemure and Mascott, 1995). There were no peri- or post-operative complications. Hemiplegia was transiently aggravated immediately after surgery. Later, the patient had no more seizures, epilepsia partialis continua disappeared and the anti-epileptic drug treatment was limited to valproic acid alone. The patient is currently walking with a slight right hemiparesis, and is progressively regaining some function of the proximal part of her right arm. She is also recovering some speech, comprehension and oral expression.

RE patient no. 6 (female, age of 15 years) underwent functional hemispherotomy on the right side using the same surgical procedure. She had a history of partial seizures since the age of 10 years, and of progressive left hemiparesis with cognitive deterioration from the age of 14. Leftsided epilepsia partialis continua started six months before surgery and was refractory to conventional AEDs as well as to high-dose corticosteroids. The surgical outcome was excellent. When evaluated one year later, the patient was seizure-free on carbamazepine alone. Walking was possible without assistance, and cognitive functions were improved.

### Statistical Analysis

For comparing quantitative variables among the different groups of epilepsy patients (Figs. 1 and 4), the non-parametric Kruskal–Wallis test was used. Pairwise

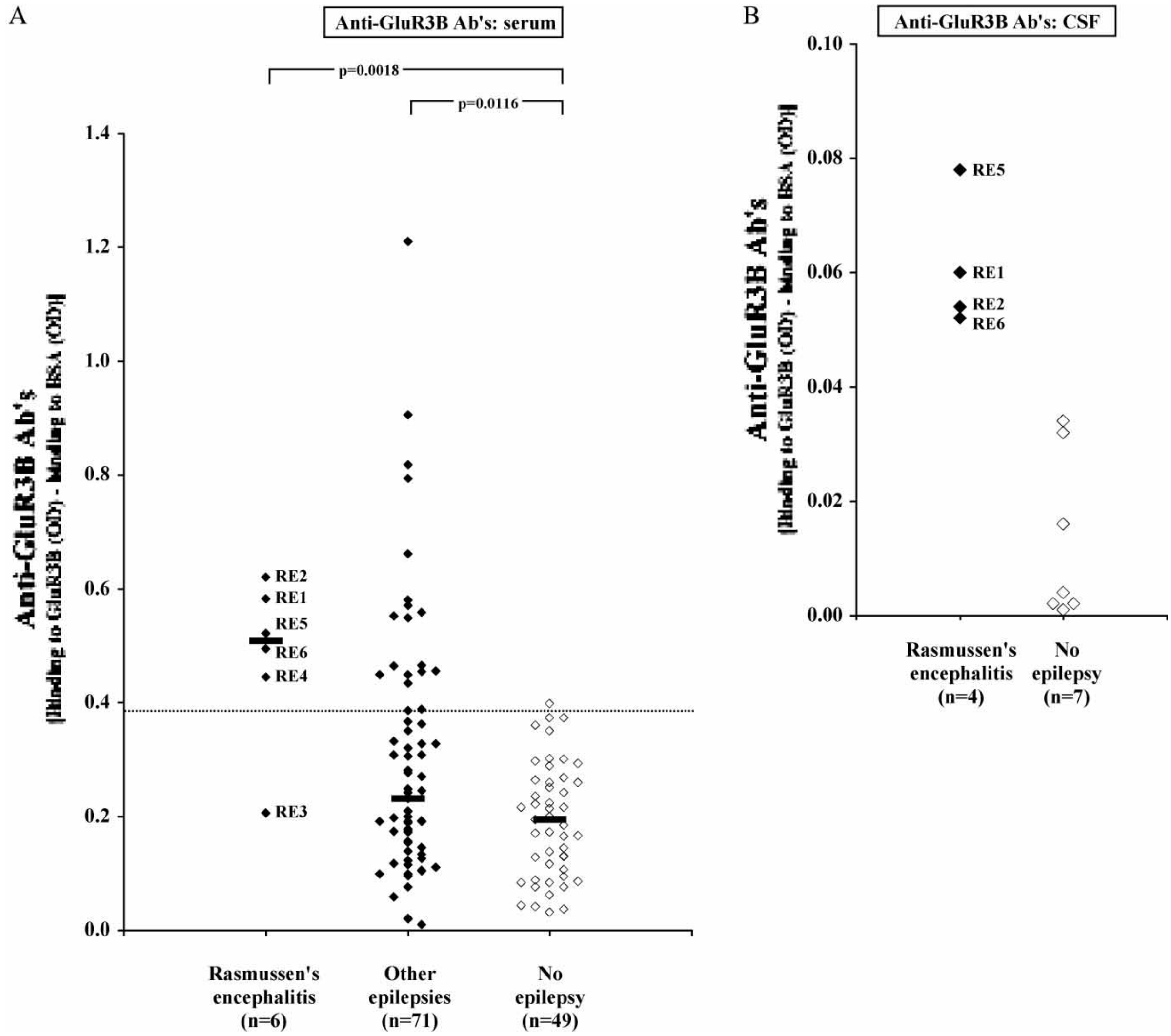

FIGURE 1 Epilepsy patients with RE or other types of epilepsy harbor significantly elevated levels of anti-GluR3B Ab's. Anti-GluR3b Ab's were measured in the sera of six RE patients and of 71 patients with other types of epilepsy (A, 1:100 dilution) and in CSF of four RE patients (B, 1:10). The horizontal lines represent the median values. Serum anti-GluR3B Ab levels were considered elevated when exceeding the mean + 2*SD of the anti-GluR3B Ab's levels in 49 neurologically healthy controls (dashed line). Numbers (RE1–RE6) relate to individual RE patients. Statistical analysis: (A) KWstatistic = 19.937, $p = 0.0005$, Kruskal–Wallis test; pairwise comparisons were performed with Mann-Whitney $U$ test, the $p$ values presented in the figure; (B) RE vs. controls $U -$ statistic = 0.0, $p = 0.0061$, Mann-Whitney $U$-test.

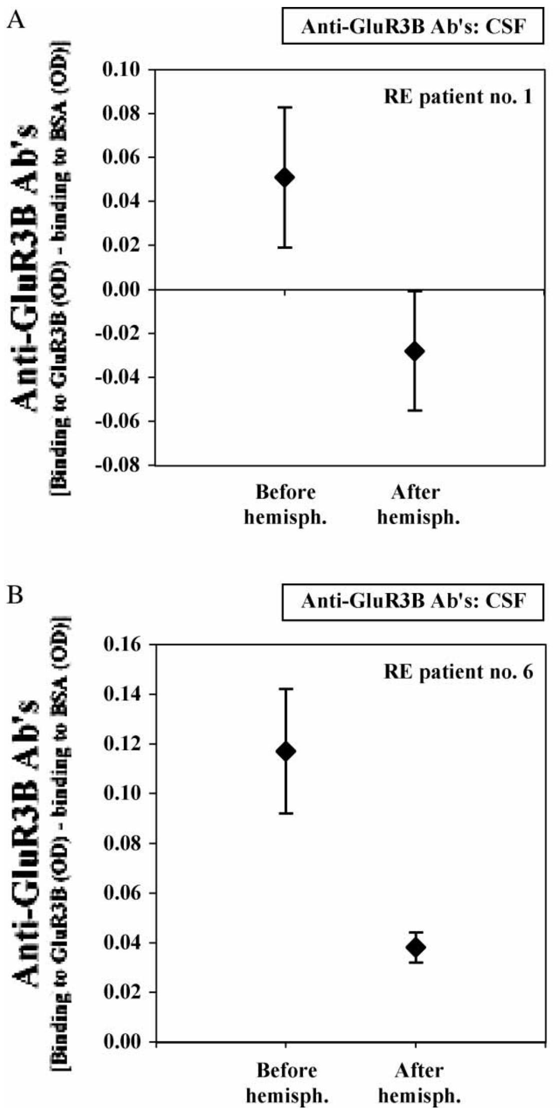

FIGURE 2 Anti-GluR3B Ab's decrease in the CSF of two RE patients after hemispherotomy. Mean ± SD of anti-GluR3B Ab's in CSF (1:10 dilution) of RE patients no. 1 (A) and 6 (B), before and after functional hemispherotomy. The corresponding neurological condition of these RE patients before and after the hemispherotomy appears in the methods.

comparisons were performed by the non-parametric Mann-Whitney $U$-test (the respective $p$-values reflect Bonferrani corrections). Formal statistical analysis of the results illustrated in Figs. 2 and 3B could not be performed due to a small number of samples. The clinical evaluation of the results presented in Fig. 5A–F (using commercially available diagnostic kits) was based on the manufacturer's guidelines and not on formal statistical analysis. All the statistical analysis was carried out using Macintosh InStat-GraphPad Software, Version 2.01.

The statistical analysis was performed in collaboration and under the instruction of Professor David Steinberg, head of the department of Statistics and Operations Research, School of Mathematical Sciences, Tel Aviv University, and of Dr. Pnina Lilos, Tel Aviv University.

### RESULTS

### Anti-GluR3B Ab's are Present on both Sides of the Blood–brain Barrier (BBB) of RE Patients, and in the Serum of some Patients with other Types of Epilepsy

We tested for specific anti-GluR3B Ab's in epilepsy patients by measuring, in parallel, specific binding of serum/CSF to the GluR3B peptide (aa 372–395 of the ionotropic glutamate receptor of the AMPA subtype 3) and nonspecific binding to BSA. The final value of specific anti-GluR3B Ab's was calculated for each individual separately as (specific-binding to GluR3B—nonspecific-binding to BSA). Using this methodology we found significantly elevated levels of specific anti-GluR3B Ab's in the sera of five of six RE patients, compared to 49 neurologically healthy control individuals (Fig. 1A). The difference between the anti-GluR3B Ab levels in the RE patients and controls was statistically significant ($U -$ statistic $= 23.00$, $p = 0.0018$; Mann-Whitney $U$-test). Elevated levels of anti-GluR3B Ab's were also detected in the sera of 17 of 71 (24%) patients with other types of epilepsy (see "Methods" section; $U -$ statistic $= 1222.0$, $p = 0.0116$; Mann-Whitney $U$-test), confirming and extending the notion that anti-GluR3B Ab's are neither specific nor restricted to RE (Wiendl *et al.*, 2001; Mantegazza *et al.*, 2002).

If indeed anti-GluR3 Ab's play a role in epilepsy, one ought to find them within the CNS, and not only in the serum. Testing the CSF samples available from only four RE patients, we found that all had significantly elevated CSF levels of anti-GluR3B Ab's, as compared to seven control CSF samples (Fig. 1B, $U -$ statistic $= 0.0$, $p = 0.0061$; Mann-Whitney $U$ test). Thus, the RE patients studied herein have anti-GluR3B Ab's on both sides of their BBB.

### Is there an Intrathecal Production of Ab's in RE Patients?

In principle, Ab's found in brain fluids of patients are either produced intrathecally or ooze from blood into the brain fluids across a leaky or damaged BBB. To investigate the possible intrathecal production of Ab's in the RE patients found positive for anti-GluR3B Ab's in the CSF (Fig. 1B), we adopted the commonly-accepted calculation of the IgG index $=$ (CSF IgG/serum IgG)/(CSF albumin/serum albumin). An IgG index value $> 0.7$ is indicative of an intrathecal Ab production (Link and Tibbling, 1977; Lefvert and Link, 1985). The IgG index was calculated only for three of the four RE patients, from whom CSF samples were available.

The results showed that RE patient no. 1 (RE1, Fig. 1B) had an IgG index value of 2.6 at the onset of the disease, and of 1.3 a year later, suggesting an intrathecal production of Ab's (regardless of their specificity). In contrast, RE patients no. 2 and 6 (RE2 and RE6, Fig. 1B) had IgG index values of 0.52 and 0.62

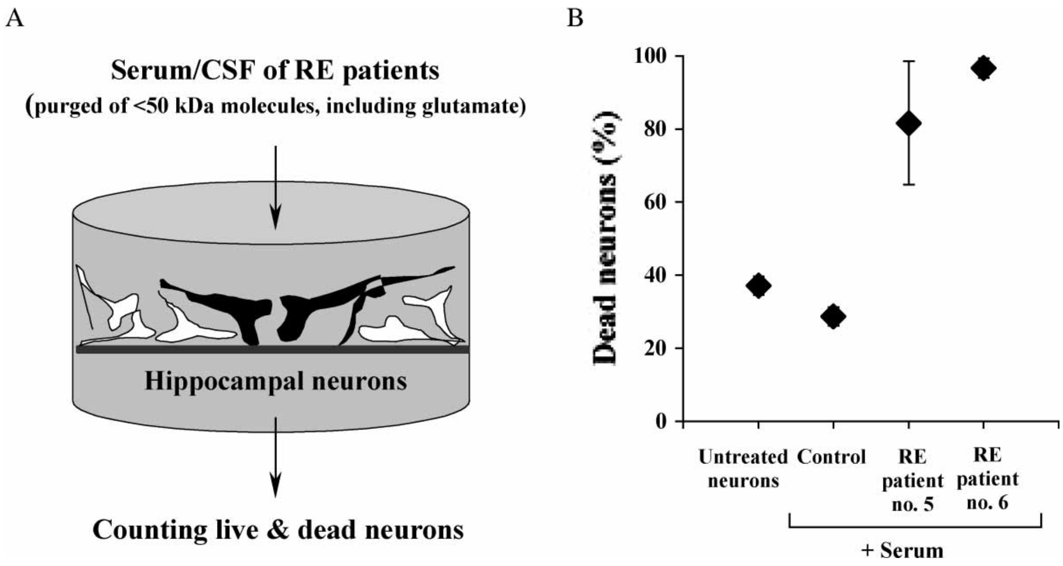

FIGURE 3 The serum of some RE patients kills neurons. (A) Cultures of rat hippocampal neurons were either left untreated, or exposed to serum (1:10 dilution) of anti-GluR3B positive RE patients no. 5 and 6, and of neurologically healthy controls, after being purged of $<$ 50 kDa molecules. The results (B) represent the mean percentage $\pm$SD of dead hippocampal neurons in two independent experiments, using independent neuronal cultures; 500–800 neurons were counted per coverslip, and two coverslips were analyzed for each sample.

respectively, arguing against an intrathecal Ab's production. Thus, in some RE patients there is an indication for intrathecal Ab production, while in others not.

### Anti-GluR3B Ab's Levels Decrease in the CSF of Two RE Patients after Beneficial Hemispherotomy

We reasoned that if the seizures in RE patients may be genuinely associated either directly or indirectly with the presence of anti-GluR3B Ab's in the CSF, the seizure decline/arrest after functional hemispherotomy, often observed in RE patients, should be accompanied by a parallel decrease in CSF anti-GluR3B Ab levels. To test this hypothesis, we compared the levels of anti-GluR3B Ab's in the CSF of two RE patients before and after hemispherotomy. For both RE patients (no. 1 and 6), the functional hemispherotomy was accompanied by seizure arrest and by a marked neurological improvement (see "Methods" section). Herein, we found that in both cases, the hemispherotomy was also followed by a dramatic reduction of anti-GluR3B Ab's in the CSF (Fig. 2). Possible reasons for such a decrease in the levels of anti-GluR3B Ab's in brain fluids after hemispherotomy are raised in the discussion.

### Serum and CSF of some RE Patients Kill Hippocampal Neurons

Neuronal death is a major feature underlining the brain pathology of RE patients (Rasmussen *et al.*, 1958). To test the possibility that anti-GluR3B Ab's (and possibly other Ab's) found in RE patients may kill neurons, as we previously found for mouse anti-GluR3B Ab's (Levite *et al.*, 1999), we measured the extent of neuronal cell death upon exposure of cultured hippocampal neurons to sera and CSF of anti-GluR3B Ab-positive RE patients, and to sera and CSF of controls (Fig. 3A). Of note, before their addition to the neuronal cultures, the sera/CSF were purged from small molecules such as glutamate, in order to eliminate their possible contribution to neuronal death and retain only $>$ 50 kDa high molecular weight molecules alike IgGs.

We found that the sera of RE patients no. 5 and 6 killed 82 and 97% of the cultured hippocampal neurons, respectively, compared to 37% death in untreated cultures, and 29% death in cultures exposed to control sera (Fig. 3B). Of note, the only common Ab's shared by RE patients no. 5 and 6 are the anti-GluR3B Ab's (see the summary of the findings in Fig. 5J). Importantly, the pre-hemispherotomy CSF of RE patient no. 6 also consistently killed 50–55% neurons, whereas the control CSF in all experiments, but one, had no neurotoxic effect (data not shown). Due to insufficient amounts of post-hemispherotomy CSF from RE patient no. 6, we could unfortunately not evaluate its ability to kill neurons.

The tests of the neuropathogenic potential of the sera and CSF of the other RE patients led to inconclusive results, inasmuch as some samples induced neuronal cell death while others did not. This could be due to the fact that samples were withdrawn from patients at different time points, under the influence of different medications, or for unknown reasons.

### Anti-dsDNA Ab's are Found on both Sides of the BBB of some RE Patients, and in the Sera of Patients with other Types of Epilepsy

Do RE patients harbor, in addition to anti-GluR3B Ab's, other potentially-pathogenic autoimmune Ab's found characteristically in "classical" autoimmune diseases? For the several reasons listed below, we focused first our

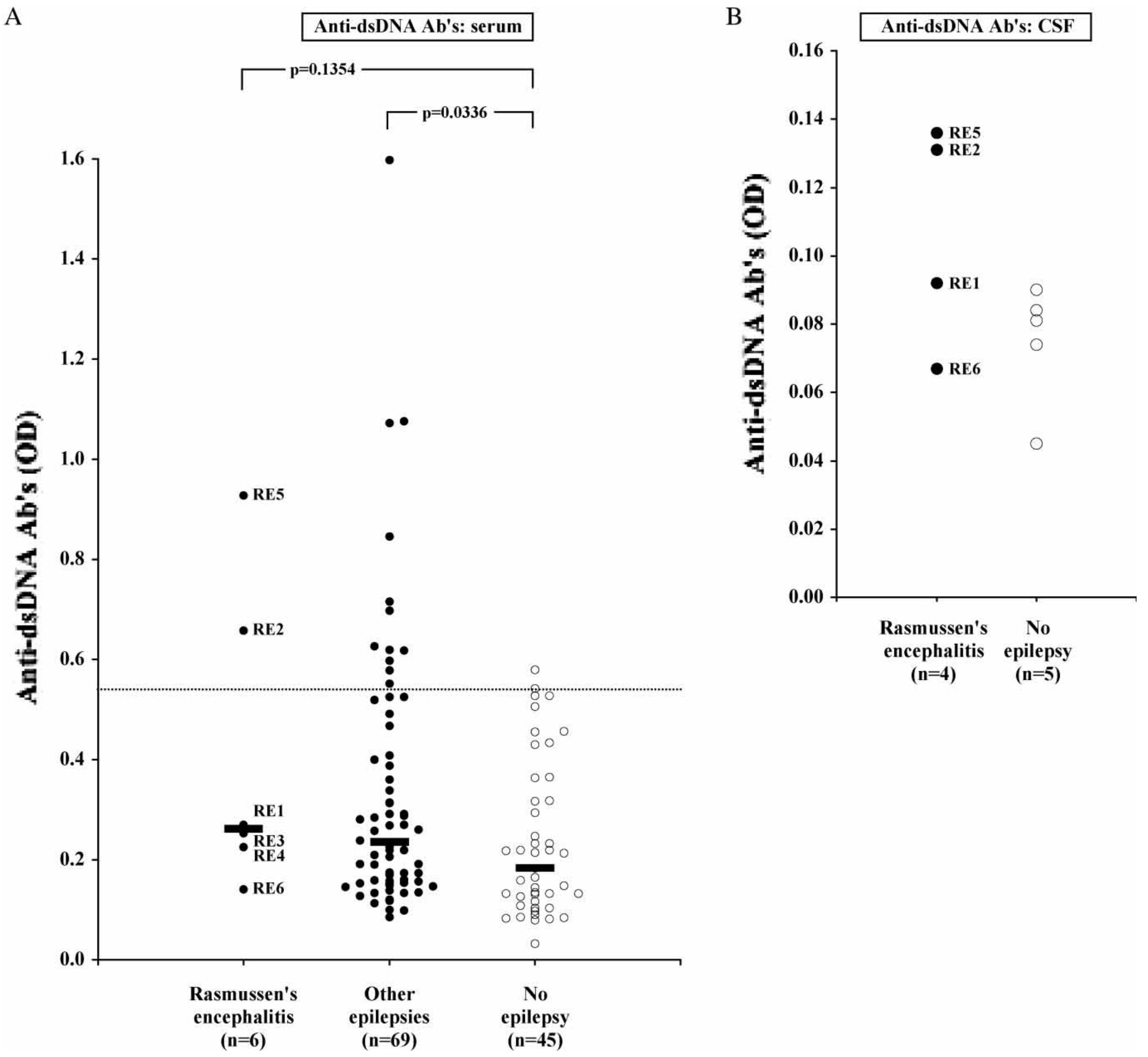

FIGURE 4 Epilepsy patients with RE or other types of epilepsy harbor significantly elevated levels of anti-dsDNA Ab's. Anti-dsDNA Ab's were measured in sera of six RE patients and of 69 patients with other types of epilepsy (A, 1:100 dilution) and in CSF of four RE patients (B, 1:10). The results present the mean OD of three independent experiments (horizontal lines- median values). Serum levels were considered elevated when exceeding the mean + 2*SD of anti-dsDNA Ab level in 45 neurologically healthy controls (dashed line). RE = Rasmussen's encephalitis, numbers relate to individual patients. Statistical analysis: (A) KW-statistic = 20.578, $p = 0.0004$, Kruskal–Wallis test; pairwise comparisons were performed with the Mann-Whitney *U*-test, the *p* values presented in the figure; (B) Comparing the RE group to the control group showed that the levels of anti-dsDNA Ab's in the CSF were not statistically different (*U*-statistic = 4.0000, $p = 0.1905$, Mann-Whitney *U*-test), but elevated levels of anti-dsDNA Ab's were detected in two RE patients (no. 5 and 2).

attention on anti-dsDNA Ab's, which are the hallmark of SLE and are known to cause immune complexes and tissue damage (Hahn, 1998). Interestingly, an RE patient who developed SLE was recently reported (Lascelles *et al.*, 2002), and from the "other side of the coin": 7–8% of SLE patients suffer from epilepsy (and other neurological complications) (Toubi *et al.*, 1995; Sanna *et al.*, 2003). We also previously observed that mice immunized with the GluR3B peptide developed not only the corresponding anti-GluR3B Ab's, but also anti-DNA Ab's (Levite and Hermelin, 1999; Levite *et al.*, 1999).

Testing herein the sera of six RE patients and of 69 patients with other types of epilepsy, we found significantly elevated levels of anti-dsDNA Ab's in the sera of two RE patients, and in the sera of 12 of 69 (17%) patients with other epilepsies (Fig. 4A). Importantly, the two RE patients, no. 5 and 2, found with elevated levels of anti-dsDNA Ab's in their serum, also harbored elevated levels of such Ab's in their CSF (Fig. 4B). To the best of our knowledge, this is the first demonstration of anti-dsDNA Ab's in the serum, and more importantly in brain fluids of epilepsy patients.

### The Sera of RE Patients contain Additional Potentially-pathogenic Ab's Typical of "Classical" Autoimmune Diseases

Table I summarizes the additional autoimmune Ab's tested in this study, the specific autoantigens they recognize, the autoimmune diseases in which they are characteristically found, and some of their known pathogenic activities in peripheral organs and in the nervous system of humans and/or animal models.

TABLE I Characteristic features and pathological function of specific Ab's found in "classical" autoimmune diseases and in some epilepsy patients

| Autoantibody | Antigen function | Found primarily in | Found also in | Pathological function of autoantibodies | References |
|---|---|---|---|---|---|
| Anti-glutamate receptor (GluR3) | Ion-channel coupled receptor for the excitatory neurotransmitter, glutamate. | Rasmussen's encephalitis | Noninflammatory focal epilepsy and "Catastrophic" epilepsy | 1. Kill neurons and astrocytes. 2. Cause epilepsy in rabbits. 3. Cause inflammatory histopathology in rabbits and mice. | Rogers *et al.* (1994), Levite *et al.* (1999), Levite and Hermelin (1999), Whitney and McNamara (2000) and Koustova *et al.* (2001) |
| Anti-dsDNA | DNA | Systemic lupus erythematosus (SLE) | | May cause damage by forming immune complexes in different tissues, or by penetrating into cells. | Hahn (1998) |
| Anti-GAD | Biosynthetic enzyme which converts glutamate into GABA. | Insulin-dependent diabetes mellitus (IDDM), Stiff-person syndrome (SPS) | Chronic cerebellar ataxia, Drug-resistant epilepsy, Myoclonus | Can suppress GABA-mediated transmission and reduce GABA synthesis in nerve terminals. | Vianello *et al.* (2002) |
| Anti-Cardiolipin | A phospholipid, which is an important component of the internal mitochondrial membrane, critical for mitochondrial function, and is involved in the pathway for cytocrome C release. | Anti-phospholipid syndrome (APS) and other thrombotic disorders, SLE | Non-thrombotic neurological disorders such as chorea/epilepsy, Recurrent fetal loss | May contribute to tissue damage by forming immune complexes, interfering with the coagulation cascade and increasing the risk of vascular complications. | Luzzana *et al.* (2002) |
| Anti-β2 glyco-protein-I | A plasma globulin involved in blood coagulation, a co-factor for binding cardiolipin. | APS and other thrombotic disorders | Fetal loss in the context of SLE | 1. May contribute to tissue damage by forming immune complexes and increasing the risk of thrombocytopenia. 2. Induce hyperactive behavior and learning/memory deficits in mice. 3. Induce depolarization of brain synaptoneurosomes. | Shoenfeld *et al.* (1998), George *et al.* (1999) and Katzav *et al.* (2003) |
| Anti-RNP | Nuclear proteins of 68 kDa, 33 kDa and 22 kDa, which are part of the U1-small nuclear ribonucleoprotein (RNP) complex, and are importanat in pre-mRNA splicing. | Mixed-connective tissue disease, SLE, | Sjögren's Syndrome and Systemic sclerosis | May cause apoptotic death of lymphocytes. | Alarcon-Segovia *et al.* (1996) and Hiepe *et al.* (2000) |
| Anti-SS-A | SS-A is composed of 52 kDa and 60 kDa RNPs, containing small uridine-rich nucleic acids. The 52 kDa RNP is a DNA-binding protein. The 60 kDa RNP is involved in the quality control pathway for 5S rRNA production and in translation of ribosomal protein mRNA (60 kDa). | Sjögren's Syndrome, SLE | Neonatal/subacute cutaneous SLE, Rheumatoid arthritis | 1. Induce conduction abnormalities/block in human fetal heart and in hearts of neonatal and adult rabbits/mice. 2. May cross-react with the serotoninergic 5HT receptor. | Eftekhari *et al.* (2000) and Mavragani *et al.* (2000) |

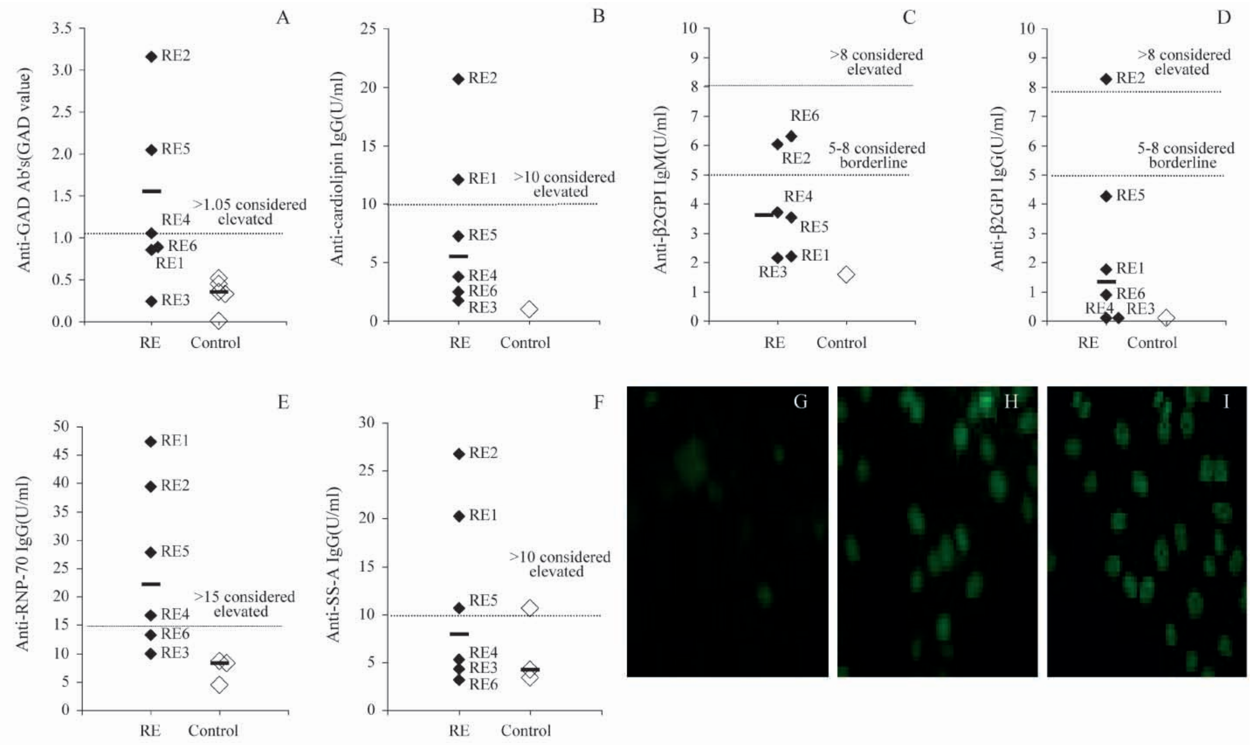

J

| RE patients | Patient 1 | | Patient 2 | | Patient 3 | | Patient 4 | | Patient 5 | | Patient 6 | |
|---|---|---|---|---|---|---|---|---|---|---|---|---|
| **A. Clinical information** | | | | | | | | | | | | |
| Age at sample withdrawal (years) | 3.5 | | 7 | | 4.5 | | 17 | | 15 | | 15 | |
| Age of RE diagnosis (years) | 3.5 | | 6 | | 3 | | 9 | | 9 | | 15 | |
| RE confirmed or suspected | Confirmed | | Confirmed | | Suspected | | Confirmed | | Confirmed | | Confirmed | |
| Diseases of patients prior to RE diagnosis | Streptococcus infection prior to diagnosis, herpes infection on admission. | | None | | None | | Possible viral infection (febrile illness) prior to diagnosis. | | None | | Viral infection (hepatitis) prior to diagnosis. | |
| Treatment: | | | | | | | | | | | | |
| Antiepileptic drugs | Low - moderate response | | Moderate response | | No response | | Low - moderate response | | Variable response | | No response | |
| Immunosupressive drugs | Not administered | | Good response | | No response | | Low - moderate response | | Not administered | | No response | |
| Plasmapheresis | Not performed | | Not performed | | No response | | No response | | No response | | Not performed | |
| IVIg | Transient improvement | | No response | | No response | | No response | | No response | | Not performed | |
| Hemispherotomy | Good response | | Not performed | | Not performed | | Not performed | | Not performed | | Excellent | |
| **B. Findings** | | | | | | | | | | | | |
| Autoantibodies: | Serum | CSF | Serum | CSF | Serum | CSF | Serum | CSF | Serum | CSF | Serum | CSF |
| Anti-GluR3B (IgG) | + | + | + | + | - | Not available | + | Not available | + | + | + | + |
| Anti-dsDNA (IgG) | - | - | + | + | - | | - | | + | + | - | - |
| Anti-GAD (IgG) | - | - | + | - | - | | + ? | | + | - | - | - |
| Anti-cardiolipin (IgG) | + | - | + | - | - | | - | | - | - | - | - |
| Anti-β2-glycoprotein I (IgM) | - | - | + | - | - | | - | | - | - | + | - |
| Anti-β2-glycoprotein I (IgG) | - | - | + | - | - | | - | | - | - | - | - |
| Anti-RNP (IgG) | + | - | + | - | - | | + | | + | - | - | - |
| Anti-SS-A (IgG) | + | - | + | - | - | | - | | + | - | - | - |

FIGURE 5 Some RE patients have additional potentially-pathogenic autoimmune ab's in their serum. (A–F) Levels of anti-GAD IgG (A), anti-cardiolipin IgG (B), anti-β2GPI IgM (C) and IgG (D), and anti-nuclear antigens RNP-70 IgG (E) and SS-A IgG (F) in sera of six RE patients. The levels of all the above Ab's were measured with commercially-available diagnostic kits commonly used in hospitals worldwide (see "Methods" section). Negative and positive controls and threshold values were supplied by the manufacturers (see "Methods" section). The results represent the mean concentration of the respective Ab in serum (1:100 dilution) of one experiment, out of total three performed (horizontal lines- median values). Ab levels were considered clinically elevated when exceeding the numeric clinical threshold indicated by the manufacturer (dashed lines). (G-I) Immunofluorescence staining of HEp-2 cells for visual confirmation of ANA: serum of ANA-positive RE patient no. 1 (H), ANA-negative RE patient no. 3 (G), and ANA-positive control included in the diagnostic kit (I). (J) Summary of clinical information regarding the six RE patients included in this study and of the findings regarding the various Ab's detected in their serum and CSF.

The results obtained herein for the six RE patients are summarized in Fig. 5J.

In specific, we found that the sera of some RE patients contain elevated levels of anti-GAD IgG (Fig. 5A), anti-cardiolipin IgG (Fig. 5B), anti-β2GPI IgM and IgG (Fig. 5C, D), and anti-nuclear (ANA) IgG's directed against RNP-70 (Fig. 5E) and SS-A (Fig. 5F). The presence of ANA in the serum was further confirmed by nuclear immunostaining of fixed HEp-2 cells (Fig. 5G–I). It is noteworthy that autoimmune Ab's of the IgG isotype, as found in the RE patients, are found characteristically only in patients at the progressive stages of the respective autoimmune disease, often in good correlation with the clinical status of the patients (e.g. see Carreras *et al.* (2000)).

No clinically significant levels of Ab's directed against either GAD, cardiolipin, β2GPI or nuclear antigens were detected in the CSF of the four RE patients tested (data not shown).

### DISCUSSION

The salient findings of the present study are as follows: (1) 29% (22 of 77) of patients with different types of epilepsy have significantly elevated levels of anti-GluR3B Ab's in serum. These results show that anti-GluR3B Ab's are more frequent among epilepsy patients than originally assumed, and that they are neither restricted nor specific to RE. This notion is in line with previous studies (Wiendl *et al.*, 2001; Mantegazza *et al.*, 2002). Importantly, anti-GluR3B Ab's may be present both in the serum and in brain fluids (as observed herein for four RE patients). Thus, some patients with epilepsy may harbor anti-GluR3B Ab's on both sides of their BBB; (2) Following functional hemispherotomy (performed to two of the RE patients studied herein), anti-GluR3B Ab levels in the CSF may drop markedly, together with seizure arrest and neurological improvement. These findings suggest that in epilepsy patients undergoing functional hemispherotomy, the high seizure frequency and impaired neurological condition pre-surgery, and the improvement in both features post surgery, may be somehow related to the level of anti-GluR3B Ab's in the CSF before and after this brain operation (i.e. high and low, respectively); (3) Sera and CSF of RE patients rich in anti-GluR3B Ab's can kill hippocampal neurons. If anti-GluR3B Ab's indeed do so *in vivo* (which remains to be shown directly), this may certainly add to the overall pathology observed in brains of RE patients; (4) 19% (14 of 75) of patients with different types of epilepsy contain significantly elevated levels of anti-dsDNA Ab's. Such anti-dsDNA Ab's may be found both in serum and in brain fluids (as found herein for two RE patients). Characteristically, elevated levels of anti-dsDNA Ab's, which are known to cause tissue damage, are found almost exclusively in serum of SLE patients. To the best of our knowledge, this is the first demonstration of elevated anti-dsDNA Ab's within the serum, and more importantly brain fluids, of patients with epilepsy; (5) Some epilepsy patients, as found in this study for the RE patients, may also contain clinically elevated levels of anti-self IgG's, directed against GAD, cardiolipin, β2GPI, RNP-70 and SS-A. All these autoimmune Ab's can exert pathogenic effects once present in the brain or in the peripheral organs (see Table I).

Collectively, all the above findings suggest that some epilepsy patients may have a kaleidoscope of potentially detrimental anti-self Ab's on both sides of the BBB, which in principle could contribute to the epilepsy and the associated brain pathology. These observations and suggestions are in line with the findings of other anti-self Ab's, such as anti-Munc 18 Ab's (Yang *et al.*, 2000) and anti-NMDA receptor Ab's, in the serum and CSF of patients with epilepsia partialis continua (EPC)/RE (Takahashi *et al.*, 2003).

A still unresolved issue is what causes the production of the anti-self Ab's in some of the epilepsy patients. At present, we can only speculate that this autoimmunity might be a consequence of either a specific or a non-specific "irritation" of the immune system, for example by certain viruses, bacteria, vaccinations in childhood, food poisoning, injury, trauma, stress or any other condition. Such a speculation is strengthened to some extent by a recent study (Takahashi *et al.*, 2003) claiming that the "causative or underlying factors" of chronic EPC in 15 patients were infection in five, vaccination in three, encephalitis in two, and head trauma in one.

In principle, the presence of anti-GluR3 Ab's in the CSF of epilepsy patients could result from an intrathecal production (as suggested by the IgG index of RE patient no. 1) or from a translocation of such Ab's from the plasma, via a leaky BBB (as suggested by the IgG index of RE patients no. 2 and 6). Of note, a recent study finds no evidence for intrathecal synthesis of anti-GluR3A (GluR3 aa 274–302) IgG in four RE patients (Mantegazza *et al.*, 2002).

Another intriguing question concerns the mechanism by which the level of anti-GluR3 Ab's decrease in the CSF after functional hemispherotomy (as shown here for RE patients no. 1 and 6). Currently and retrospectively, there is no way to answer this question. Yet, based on the common knowledge that maintenance of high levels of Ab's (regardless of their type, site of production and context) requires an active ongoing production of these Ab's by a potent immune system, and a steady supply of the respective antigen, one can speculate that the hemispherotomy might have decreased either one or even both of these processes. Thus, the brain surgery might have weakened the overall state of the immune system in a nonspecific manner (i.e. caused a transient immunodepression), and/or might have reduced the availability of the free GluR3B antigen (for instance by down regulating the enzymatic cleavage of the short GluR3B peptide from its parent receptor: GluR3 (Gahring *et al.*, 2001)). Of note, we recently found that full length GluR3 is expressed not only on neurons and glia, but also in high levels on human T-cells (Ganor *et al.*, 2003). On top of these two hypothetical reasons which might have caused the drastic decrease of anti-GluR3B Ab's after the hemispherotomy, one could suggest an additional, more trivial explanation, whereby the drainage of CSF during surgery may have diluted the anti-GluR3B Ab's. Of note, by discussing our findings and suggestions regarding the levels of anti-GluR3B Ab's before and after hemispherectomy, we by no means claim that this is the most important factor responsible for the seizure arrest. Clearly, the most crucial factor for the post-surgery improvement is the elimination of the seizure loci. Yet, the possibility that additional autoimmune factors, and specifically the decrease in the levels of anti-GluR3B Ab's within the brain fluids, might have contributed to the beneficial outcome, deserves attention in future studies.

Neuronal death is a characteristic feature in RE (Rasmussen *et al.*, 1958). Herein, we demonstrate that sera and CSF of some epilepsy patients (e.g. RE patients no. 5 and 6), which contain elevated levels of anti-GluR3B Ab's, have the ability to kill hippocampal neurons. This is in line with the previous observations by others and ourselves that both mouse anti-GluR3B Ab's and rabbit anti-GluR3 Ab's (directed against the 212aa long extracellular fragment) kill neurons and cause an inflammatory histopathology (Rogers *et al.*, 1994; He *et al.*, 1998; Levite and Hermelin, 1999; Levite *et al.*, 1999; Whitney and McNamara, 2000; Koustova *et al.*, 2001). Yet, the precise type and nature of the human Ab's causing neuronal death, the mechanism by which the latter takes place (i.e. exitotoxicity or complement fixation or other), and whether the Ab-induced neuronal death leads necessarily to epilepsy are all still open questions which await further investigations, preferably to be carried out in several research centers worldwide, and on a much larger number of patients.

Finally, despite all the above open issues, even at present we advise to test for the Ab's studied herein in epilepsy patients, especially when confronting severe intractable patients. We further recommend that upon finding patients with significantly elevated levels of autoimmune Ab's, some modes of immunotherapy should be considered (for review see Levite and Hart (2002)), (Rogers *et al.*, 1994; Andrews *et al.*, 1996; Antozzi *et al.*, 1998; Granata *et al.*, 2003; Takahashi *et al.*, 2003), preferentially before drastic brain surgery, and perhaps concomitantly with classical AEDs.

### Acknowledgements

The authors thank Professor Zvi Vogel, head of the Department of Neurobiology, The Weizmann Institute of Science, and Professor Dov Lichtenberg, Dean of the Sackler Faculty of Medicine, Tel-Aviv University, for their important support and positive attitude that enabled this study to be performed. We also thank warmly Professor Olivier Dulac (Paris), for a critical reading of the manuscript and for his valuable and incisive comments, and Professor Edna Mozes for introducing us, together with Heidi Zinger, to the method of detection of dsDNA-specific Ab's. Special gratitude and appreciation to Professor David Steinberg, head of the department of Statistics and Operations Research, School of Mathematical Sciences, Tel Aviv University, for his rigorous statistical analysis and for his didactic comments.

This study was supported by international grants to Dr M. Levite.

### References

Alarcon-Segovia, D., Llorente, L. and Ruiz-Arguelles, A. (1996) "The penetration of autoantibodies into cells may induce tolerance to self by apoptosis of autoreactive lymphocytes and cause autoimmune disease by dysregulation and/or cell damage", *J. Autoimmun.* **9**, 295–300.

Andrews, P.I., Dichter, M.A., Berkovic, S.F., Newton, M.R. and McNamara, J.O. (1996) "Plasmapheresis in Rasmussen's encephalitis", *Neurology* **46**, 242–246.

Antozzi, C., Granata, T., Aurisano, N., *et al.* (1998) "Long-term selective IgG immuno-adsorption improves Rasmussen's encephalitis", *Neurology* **51**, 302–305.

Bernasconi, P., Cipelletti, B., Passerini, L., *et al.* (2002) "Similar binding to glutamate receptors by Rasmussen and partial epilepsy patients' sera", *Neurology* **59**, 1998–2001.

Brann, A.B., Tcherpakov, M., Williams, I.M., Futerman, A.H. and Fainzilber, M. (2002) "Nerve growth factor-induced p75-mediated death of cultured hippocampal neurons is age-dependent and transduced through ceramide generated by neutral sphingomyelinase", *J. Biol. Chem.* **277**, 9812–9818.

Carreras, L.O., Forastiero, R.R. and Martinuzzo, M.E. (2000) "Which are the best biological markers of the antiphospholipid syndrome?", *J. Autoimmun.* **15**, 163–172.

Choi, D.W. (1992) "Excitotoxic cell death", *J. Neurobiol.* **23**, 1261–1276.

Eftekhari, P., Salle, L., Lezoualc'h, F., *et al.* (2000) "Anti-SSA/Ro52 autoantibodies blocking the cardiac 5-HT4 serotoninergic receptor could explain neonatal lupus congenital heart block", *Eur. J. Immunol.* **30**, 2782–2790.

Eilat, E., Dayan, M., Zinger, H. and Mozes, E. (2001) "The mechanism by which a peptide based on complementarity-determining region-1 of a pathogenic anti-DNA auto-Ab ameliorates experimental systemic lupus erythematosus", *Proc. Natl Acad. Sci. USA* **98**, 1148–1153.

Frassoni, C., Spreafico, R., Franceschetti, S., *et al.* (2001) "Labeling of rat neurons by anti-GluR3 IgG from patients with Rasmussen encephalitis", *Neurology* **57**, 324–327.

Gahring, L., Carlson, N.G., Meyer, E.L. and Rogers, S.W. (2001) "Granzyme B proteolysis of a neuronal glutamate receptor generates an autoantigen and is modulated by glycosylation", *J. Immunol.* **166**, 1433–1438.

Ganor, Y., Besser, M., Ben-Zakay, N., Unger, T. and Levite, M. (2003) "Human T cells express a functional ionotropic glutamate receptor GluR3, and glutamate by itself triggers integrin-mediated adhesion to laminin and fibronectin and chemotactic migration", *J. Immunol.* **170**, 4362–4372.

George, J., Gilburd, B., Langevitz, P., *et al.* (1999) "Beta2 glycoprotein I containing immunecomplexes in lupus patients: association with thrombocytopenia and lipoprotein (a) levels", *Lupus* **8**, 116–120.

Granata, T., Fusco, L., Gobbi, G., *et al.* (2003) "Experience with immunomodulatory treatments in Rasmussen's encephalitis", *Neurology* **61**, 1807–1810.

Hahn, B.H. (1998) "Antibodies to DNA", *N. Engl. J. Med.* **338**, 1359–1368.

He, X.P., Patel, M., Whitney, K.D., Janumpalli, S., Tenner, A. and McNamara, J.O. (1998) "Glutamate receptor GluR3 antibodies and death of cortical cells", *Neuron* **20**, 153–163.

Hiepe, F., Dorner, T. and Burmester, G. (2000) "Antinuclear antibody- and extractable nuclear antigen-related diseases", *Int. Arch. Allergy Immunol.* **123**, 5–9.

Katzav, A., Chapman, J. and Shoenfeld, Y. (2003) "CNS dysfunction in the antiphospholipid syndrome", *Lupus* **12**, 903–907.

Koustova, E., Sei, Y., Fossom, L., *et al.* (2001) "LP-BM5 virus-infected mice produce activating autoantibodies to the AMPA receptor", *J. Clin. Invest.* **107**, 737–744.

Lascelles, K., Dean, A.F. and Robinson, R.O. (2002) "Rasmussen's encephalitis followed by lupus erythematosus", *Dev. Med. Child Neurol.* **44**, 572–574.

Lefvert, A.K. and Link, H. (1985) "IgG production within the central nervous system: a critical review of proposed formulae", *Ann. Neurol.* **17**, 13–20.

Levite, M. (2002) "Autoimmune epilepsy", *Nat. Immunol.* **3**, 500.

Levite, M. and Hart, I.K. (2002) "Immunotherapy for epilepsy", *Expert Rev. Neurother.* **2**, 804–819.

Levite, M. and Hermelin, A. (1999) "Autoimmunity to the glutamate receptor in mice—a model for Rasmussen's encephalitis?", *J. Autoimmun.* **13**, 73–82.

Levite, M., Fleidervish, I.A., Schwarz, A., Pelled, D. and Futerman, A.H. (1999) "Autoantibodies to the glutamate receptor kill neurons via activation of the receptor ion channel", *J. Autoimmun.* **13**, 61–72.

Link, H. and Tibbling, G. (1977) "Principles of albumin and IgG analyses in neurological disorders. III. Evaluation of IgG synthesis within the central nervous system in multiple sclerosis", *Scand. J. Clin. Lab. Invest.* **37**, 397–401.

Luzzana, C., Gerosa, M., Riboldi, P. and Meroni, P.L. (2002) "Up-date on the antiphospholipid syndrome", *J. Nephrol.* **15**, 342–348.

Mantegazza, R., Bernasconi, P., Baggi, F., *et al.* (2002) "Antibodies against GluR3 peptides are not specific for Rasmussen's encephalitis but are also present in epilepsy patients with severe, early onset disease and intractable seizures", *J. Neuroimmunol.* **131**, 179–185.

Mavragani, C.P., Tzioufas, A.G. and Moutsopoulos, H.M. (2000) "Sjogren's syndrome: autoantibodies to cellular antigens. Clinical and molecular aspects", *Int. Arch. Allergy Immunol.* **123**, 46–57.

Olney, J.W. (1990) "Excitotoxicity: an overview", *Can. Dis. Wkly Rep.* **16**(Suppl. 1E), 47–57, discussion 57-8.

Proposal for revised classification of epilepsies and epileptic syndromes. Commission on Classification and Terminology of the International League Against Epilepsy. *Epilepsia* 1989; 30:389–99.

Rasmussen, T., Olszewski, J. and Lloydsmith, D. (1958) "Focal seizures due to chronic localized encephalitis", *Neurology* **8**, 435–445.

Rogers, S.W., Andrews, P.I., Gahring, L.C., *et al.* (1994) "Autoantibodies to glutamate receptor GluR3 in Rasmussen's encephalitis", *Science* **265**, 648–651.

Sanna, G., Bertolaccini, M.L., Cuadrado, M.J., *et al.* (2003) "Neuropsychiatric manifestations in systemic lupus erythematosus: prevalence and association with antiphospholipid antibodies", *J. Rheumatol.* **30**, 985–992.

Shoenfeld, Y., Gharavi, A. and Koike, T. (1998) "Beta2GP-I in the anti phospholipid (Hughes') syndrome—from a cofactor to an autoantigen—from induction to prevention of antiphospholipid syndrome", *Lupus* **7**, 503–506.

Takahashi, Y., Mori, H., Mishina, M., *et al.* (2003) "Autoantibodies to NMDA receptor in patients with chronic forms of epilepsia partialis continua", *Neurology* **61**, 891–896.

Toubi, E., Khamashta, M.A., Panarra, A. and Hughes, G.R. (1995) "Association of antiphospholipid antibodies with central nervous system disease in systemic lupus erythematosus", *Am. J. Med.* **99**, 397–401.

Twyman, R.E., Gahring, L.C., Spiess, J. and Rogers, S.W. (1995) "Glutamate receptor antibodies activate a subset of receptors and reveal an agonist binding site", *Neuron* **14**, 755–762.

Vianello, M., Tavolato, B. and Giometto, B. (2002) "Glutamic acid decarboxylase autoantibodies and neurological disorders", *Neurol. Sci.* **23**, 145–151.

Villemure, J.G. and Mascott, C.R. (1995) "Peri-insular hemispherotomy: surgical principles and anatomy", *Neurosurgery* **37**, 975–981.

Whitney, K.D. and McNamara, J.O. (2000) "GluR3 autoantibodies destroy neural cells in a complement-dependent manner modulated by complement regulatory proteins", *J. Neurosci.* **20**, 7307–7316.

Wiendl, H., Bien, C.G., Bernasconi, P., *et al.* (2001) "GluR3 antibodies: Prevalence in focal epilepsy but no specificity for Rasmussen's encephalitis", *Neurology* **57**, 1511–1514.

Xiong, Z.Q., Qian, W., Suzuki, K. and McNamara, J.O. (2003) "Formation of complement membrane attack complex in mammalian cerebral cortex evokes seizures and neurodegeneration", *J. Neurosci.* **23**, 955–960.

Yang, R., Puranam, R.S., Butler, L.S., *et al.* (2000) "Autoimmunity to munc-18 in Rasmussen's encephalitis", *Neuron* **28**, 375–383.

